# Overexpression of apolipoprotein C‐III decreases secretion of dietary triglyceride into lymph

**DOI:** 10.1002/phy2.247

**Published:** 2014-03-20

**Authors:** Fei Wang, Alison B. Kohan, H. Henry Dong, Qing Yang, Min Xu, Sarah Huesman, Danwen Lou, David Y. Hui, Patrick Tso

**Affiliations:** ^1^ Department of Pathology and Laboratory Medicine University of Cincinnati Cincinnati Ohio 45237; ^2^ Children's Hospital of Pittsburgh Rangos Research Center Pittsburgh Pennsylvania 15244

**Keywords:** ApoC‐III, apolipoproteins, dietary fat absorption, intestine

## Abstract

Apolipoprotein C‐III (apoC‐III) is not only predominantly synthesized by the liver but also by the small intestine. Because apoC‐III is secreted from the intestine on the chylomicron along with lipid absorption, we questioned whether apoC‐III might play a role in intestinal lipid absorption and/or transport. Using both wild‐type (WT) and apoC‐III transgenic (apoC‐III Tg) mice, we showed that apoC‐III Tg mice have decreased lymphatic lipid transport compared with WT mice in response to an intraduodenal infusion of radiolabeled lipid. This is associated with accumulation of radiolabeled lipids in the luminal compartment of the apoC‐III Tg mice, indicating delayed lipid uptake from the lumen. The total amount of radioactive lipids in the mucosal compartment did not differ between apoC‐III Tg and WT mice, but the lipid distribution analysis indicated a predominance of free fatty acids and monoacylglycerol in the mucosa of apoC‐III Tg mice, implying impaired esterification capacity. Thus, the mechanisms underlying the reduced lymphatic lipid transport in apoC‐III Tg mice involve both a delayed lipid uptake into enterocytes, as well as impaired esterification to form triglyceride in the mucosa. These data document a novel role for apoC‐III in the uptake, re‐esterification, and lymphatic transport of dietary lipids in the intestine.

## Introduction

Apolipoprotein C‐III (apoC‐III) is an exchangeable lipoprotein produced by both the liver and the intestine (Wu and Windmueller 1978; Haddad et al. 1986; Lenich et al. 1988). In humans, apoC‐III expression is also a crucial determinant of plasma triacylglycerol (TAG) levels. In human studies, plasma apoC‐III levels are an independent predictor of cardiovascular disease and the progression of atherosclerosis (Blankenhorn et al. 1990; Sacks et al. 2000), and in type 1 diabetics the plasma concentration of apoC‐III is elevated (Lee et al. 2003; Hokanson et al. 2006). In addition, overproduction of apoC‐III and of triacylglycerol‐rich lipoproteins (TRLs) containing apoC‐III is a common feature in patients with hypertriglyceridemia (Fredenrich et al. 1997; Cohn et al. 2004a; Zheng et al. 2010).

This relationship between apoC‐III and disease is related to its regulation of the clearance and metabolism of TRLs. In plasma, apoC‐III resides on very low‐density lipoproteins (VLDL) and chylomicrons, as well as high‐density lipoproteins (HDL) (Brown et al. 1969; Schonfeld et al. 1979; Fredenrich et al. 1997; Ginsberg and Brown 2011). ApoC‐III acts to delay the clearance of these particles from plasma both through the inhibition of their hydrolysis by lipoprotein lipase, and also by inhibiting their subsequent clearance through receptor‐mediated uptake by the liver (Kinnunen and Ehnolm 1976; Landis et al. 1987; Sehayek and Eisenberg 1991; Aalto‐Setala et al. 1992; McConathy et al. 1992; Zheng et al. 2007; Gangabadage et al. 2008). These functions of apoC‐III on TRL clearance link its expression with the hypertriglyceridemic state. In vivo, these functions of apoC‐III are reflected by hypertriglyceridemia found in apoC‐III transgenic (Tg) mice (Qu et al. 2007), and conversely, by the hypotriglyceridemia in apoC‐III knockout (KO) mice (Maeda et al. 1994).

Another emerging role for apoC‐III is its stimulation of hepatic VLDL synthesis and secretion. In both cell culture and human kinetic studies, apoC‐III has been reported to enhance the assembly and secretion of VLDL particles from the liver under lipid‐rich conditions (Cohn et al. 2004b; Sundaram et al. 2010a). In vitro studies suggest that increases in hepatic apoC‐III expression stimulate the secretion of larger VLDL by recruiting additional TAG loading onto the pre‐VLDL particle (Sundaram et al. 2010a,b). This mechanism involves the N‐terminal region of apoC‐III interacting with the hepatic lipoprotein synthesis machinery (Sundaram et al. 2010a).

Although significant progress has been made in understanding the mechanisms that govern these plasma and hepatic effects of apoC‐III, much less is known about the function of apoC‐III in the intestine. Particularly, how apoC‐III may impact the intestinal lipoprotein synthesis pathway and its potential effect on the metabolism of dietary lipid. This is an important gap in our knowledge since the intestinal lipoprotein synthesis and secretion pathway is unique from the liver VLDL pathway, and apoC‐III may therefore play a unique role there.

The lipoprotein pathway in the intestine diverges from the hepatic pathway as early as the source of fatty acyl precursors. Although the absorptive cells of the intestine take up free fatty acids (FFA) and monoacylglycerol (MAG) from the intestinal lumen (Mansbach and Gorelick 2007), the liver uses a variety of acyl precursors including circulating FFA, stored lipid, and remnant lipoprotein TAG. The intestinal enterocyte synthesizes TAG from its acyl precursors through the monoacylglycerol pathway, catalyzed primarily by monoacylglycerol acyltransferase (MGAT) activity, whereas hepatocytes are more reliant on the glycerol‐3‐phosphate (Kennedy) pathway, catalyzed in large part by diacylglycerol acyltransferase (DGAT) (Coleman 2004). In both liver and intestine, apolipoprotein B (apoB) serves as a scaffold for the nascent lipoprotein particles, with the intestine producing the truncated apoB‐48 and the liver producing apoB‐100. In both cases, apoB is cotranslationally translocated into the ER lumen during lipoprotein synthesis, where microsomal triglyceride transfer protein (MTP) adds TAG to immature apoB‐containing particles. A difference between intestine and liver is that the intestine retains the ability to secrete lipid‐poor apoB‐48, whereas the liver rapidly degrades lipid‐poor apoB‐100 (Guo et al. 2005; Xiao et al. 2011). Finally, the intestine utilizes the unique prechylomicron transport vesicle (PCTV) for the transport of prechylomicrons from the ER to the Golgi for maturation and secretion. The budding of the PCTV is rate limiting in the secretion of dietary TAG from the ER to the lymph, and the regulation of this step is critical for TAG secretion in intestine (Kumar and Mansbach 1999). In the liver, the VLDL transport vesicle (VTV) transports nascent VLDL from the ER to the Golgi and is also tightly regulated, however, the proteins involved in this regulation, and presumably their regulation, differ significantly from the proteins governing intestinal PCTV transport (Rahim et al. 2012).

Given these key differences in lipoprotein synthesis and secretion between the liver and intestine, we examined how apoC‐III functions in intestinal lipoprotein synthesis and secretion pathway, and specifically, whether overexpression of apoC‐III in the intestine increases lipoprotein secretion as it does in liver. For these studies, we used the well‐established lymph fistula mouse model, which has significant advantages for determining intestinal lipoprotein synthesis and secretion. These advantages include (1) it is an in vivo model, with intact circulatory, neural, and hormonal connections to the intestine; (2) the ability to measure the dynamic changes in lipid output continuously during a lipid infusion; (3) both chylomicron formation and lymph flow are not altered by anesthesia, since the mice are conscious during lipid infusion; (4) the lymph we collect reflects what is secreted by the intestine in response to dietary lipid, and is not obscured by lipolysis or modification that occur in the plasma compartment; and (5) mice are fed exclusively by a duodenal infusion tube to control and quantify the exact amount of lipid administered, without the confounding effects of gastric emptying.

We found that apoC‐III overexpression in the intestine inhibits dietary lipid uptake by the small intestine and the subsequent secretion of TAG into lymph in chylomicrons. This corresponds with an increase in luminal and mucosal FFA in the apoC‐III Tg mice. This finding suggests a novel role for apoC‐III in TG handling in the intestine that is quite unique from its hepatic role in VLDL assembly and secretion. In addition, apoC‐III, through this effect on intestinal TG handling, is clearly a modulator of dietary lipid absorption.

## Materials and Methods

### Animals

Transgenic mice expressing human apoC‐III in the C57BL/6J background (apoC‐III Tg mice) were obtained from Dr. Henry Dong, and originally generated by Dr. Jan Breslow (Ito et al. 1990; Aalto‐Setala et al. 1992; Qu et al. 2007). The apoC‐III Tg mice have whole‐body overexpression of human apoC‐III (with expression of human apoC‐III at approximately five times the WT amount) and have fasting plasma TG levels of 400–500 mg/dL. The animals are homozygous for the transgene, which retains the apoC‐III promoter to drive expression (which results in above average human apoC‐III expression in the same tissue distribution pattern as the endogenous mouse apoC‐III). The transgenic mice express both the endogenous mouse apoC‐III gene and the human apoC‐III gene, but have with no compensatory changes in endogenous mouse apoC‐III expression. WT C57BL/6J mice, obtained from Jackson Labs (Bar Harbor, ME), were bred and housed in our animal facility. All mice were housed on a 12‐h light/dark cycle with ad libitum access to food and water. We also analyzed a small subset of WT littermates to the apoC‐III Tg mice, and found no differences between this in‐house WT colony and the C57BL/6J WT mice received from Jackson Labs. This would suggest that all differences observed in the apoC‐III Tg mice were due to the overexpression of the human apoC‐III gene, rather than more generalized changes to gut microbiota or breeding conditions, although these factors may be tremendously important to intestinal function. Therefore, for all future experiments we utilized male age‐ and weight‐matched WT mice from Jackson Labs. All surgical procedures were approved by the University of Cincinnati Internal Animal Care and Use Committee and complied with the NIH Guide for the Care and Use of Laboratory Animals.

### Cannulation mesenteric lymph

Male apoC‐III Tg and WT mice weighing 28–30 g were used. The mice were fasted overnight with free access to water prior to placement of both the duodenal tube and lymphatic cannula. For these procedures, the mice were placed under anesthesia (ketamine, 80 mg/kg and xylazine, 20 mg/kg), and the superior mesenteric lymphatic duct was cannulated with polyvinyl chloride tubing, as described previously (Lo et al. 2008; Kohan et al. 2012). A duodenal cannula was also placed and secured by purse‐string suture. After surgery, mice received 5% glucose in saline infusion (0.3 mL/h) via duodenal cannula to compensate for fluid and electrolyte loss due to lymphatic drainage. Mice recovered overnight in Bollman restraint cages housed in a temperature‐regulated chamber maintained at 30°C.

### Lipid infusion

After overnight recovery, mice received a continuous lipid infusion of an emulsion containing 4 *μ*mol of triolein containing: 1 *μ*Ci (9,10‐^3^H[N]) triolein (Perkin Elmer, Boston, MA), 0.78 *μ*mol cholesterol, 0.87 *μ*mol phospholipid, in 1.9 *μ*mol sodium taurocholate (for a total infusion volume of 0.3 mL/h) for 6 h. This continuous infusion results in a steady state TG secretion into lymph, and facilitates comparison between the steady state achieved in the WT and the apoC‐III Tg mice. Lymph was collected on ice 1‐h prior to lipid infusion (fasting lymph), and then hourly throughout the 6‐h infusion period. Lymph was collected from 11 apoC‐III Tg mice and 12 WT mice. Lymphatic triolein recovery at each time point was calculated as a percentage of the total number of radioactive cpm infused into the intestine per hour.

### Collection of luminal contents and mucosal tissue

At the end of the 6‐h lipid infusion period, mice were sacrificed with an intraperitoneal injection of ketamine–xylazine. Both ends of the stomach, small intestine, and colon were tied with sutures to prevent leakage of the luminal contents. The small intestine was carefully further divided into four equal‐length segments (labeled no. 1–4, corresponding to the proximal to distal ends, respectively). The luminal contents of the four segments as well as the stomach and colon were collected by washing three times with 1 mL of 10 mmol/L sodium taurocholate. The luminal contents were then homogenized, and aliquots were taken for determination of radioactivity. The four small intestinal segments were cut open longitudinally and placed flat on a glass plate (luminal side up), and the mucosa was scraped with a glass slide (and these mucosal samples were labeled M1–M4, corresponding to the most proximal to distal quarters as above). The lipid in both the lumen and mucosa was extracted by the method of Folch et al. (1957). Radioactivity in aliquots of lymph, luminal contents, and mucosal homogenates (and extracted lipid) were determined by liquid scintillation counting. The amount of triolein remaining in the lumen or mucosa at the end of the experiment was calculated as a percentage of the total cpm of radioactive triolein infused during the 6‐h infusion period.

### Thin‐layer chromatographic analysis of lipid

Aliquots of both luminal contents and mucosal tissue (described above) were incubated at 70°C for 10 min after collection to inactivate endogenous pancreatic lipase (Roggin et al. 1972). Lipids were extracted by a modified Folch extraction (Folch et al. 1957). Total lipid extracted was solubilized in 2:1 vol/vol CHCl_3_:MeOH, and were loaded onto activated silica gel G plates, and the lipids were fractionated using a solvent system of petroleum ether, diethyl ether, and glacial acetic acid (75:15:1.2 vol/vol/vol). Iodine vapor was used to visualize the different lipid classes, as well as the comigrating lipid standards. The spots corresponding to triacylglycerol, diacylglycerol, monoacylglycerol, free fatty acid, and phospholipid were scraped into scintillation vials (containing 1 mL ethanol) prior to addition of Opti‐Fluor scintillation fluid (Perkin Elmer, Hebron, KY) for scintillation counting.

### Real‐time PCR

Total RNA was extracted from liver, duodenum, jejunum, and ileum using RNeasy isolation Kit (Qiagen Inc., Valencia, CA) and converted into cDNA using iScript (Bio‐rad Laboratories, Hercules, CA). Quantitative real‐time PCR was performed on a Bio‐Rad iCycler system. A typical PCR reaction (20 *μ*L) contained 10 *μ*L 2× Fast SYBR Green Master Mix (Applied Biosystems, Grand Island, NY), 1 *μ*L each of 5 *μ*mol/L forward and reverse primers, and a 1:10 dilution of cDNA. Copy numbers were normalized to cyclophilin A. The following primers were used: human apoC‐III, forward 5′‐GACCGATGGCTTCAGTTCC‐3′, reverse 5′‐GCAGGATGGATAGGCAGGT‐3′.

### Statistics

Values are expressed as mean ± SEM. The differences between the WT and apoC‐III Tg mice in their luminal and mucosal lipids were analyzed by Student's *t*‐test. The lymph flow rate and lymphatic [^3^H] triolein recovery were analyzed by two‐way analysis of variance (ANOVA) using Graph Pad Prism 5.0 (La Jolla, CA). Differences were considered statistically significant at *P* < 0.05.

## Results

### ApoC‐III mRNA expression and subsequent hypertriglyceridemic phenotype

**Figure 1:**
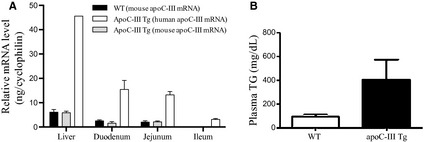
The expression of apoC‐III and plasma triglyceride levels. (A) Tissue distribution of apoC‐III mRNA in the liver and in the small intestine. In the apoC‐III Tg mice, mRNA expression of both the endogenous mouse apoC‐III (gray bars) and the transgenic expression of human apoC‐III mRNA (white bars) were measured. (B) Plasma triglycerides in WT and apoC‐III Tg mice. Values are means ± SEM,* n* = 3 in each group.

In addition to its production by the liver, the intestine also produces apoC‐III. As shown in Figure 1A, WT mouse apoC‐III mRNA is expressed most highly in liver, followed by expression at about 30% of this level along a gradient in the small intestine. This pattern of expression is maintained in the apoC‐III Tg mice, with human apoC‐III mRNA expression following the same tissue distribution, but expressed at approximately fivefold higher levels. Expression apoC‐III mRNA is highest in the proximal intestine (duodenum), which coincides with the site of highest lipid absorption (Booth et al. 1961). Endogenous mouse apoC‐III mRNA expression is not altered from WT levels in the apoC‐III Tg mice. ApoC‐III Tg mice are overtly hypertriglyceridemic, with plasma TG levels of 400–500 mg/dL (Fig. 1B).

### Overexpression of apoC‐III inhibits TG secretion into lymph

**Figure 2:**
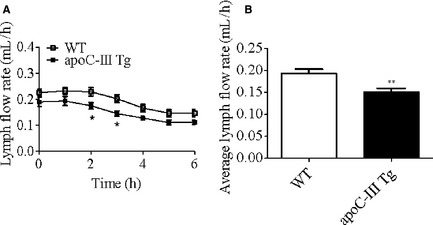
Lymph flow in WT and apoCIII Tg mice. Hourly (A) and average (B) lymph flow rates in lipid‐infused WT (*n* = 12) and apoC‐III Tg mice (*n* = 11). Mice received a continuous infusion of lipids via intraduodenal infusion tube, and lymph was collected through the intestinal lymph duct cannulation. Values are means ± SEM.*P < 0.05, **P < 0.01 versus WT mice.

Both VLDL secretion from the liver and chylomicron secretion from the intestine contribute to circulating plasma TAG levels, therefore we hypothesized that the hypertriglyceridemia in the apoC‐III Tg mice may be due to an increase in intestinal TAG absorption. To further interrogate this possibility, we utilized the lymph fistula mouse model. As shown in Figure 2A, lymph flow during continuous duodenal infusion of 4 *μ*mol/h [^3^H]‐triolein was relatively constant throughout the experiment (0.15–0.25 mL/h) for both apoC‐III Tg and WT mice. However, at both 2‐ and 3 h into the infusion period, apoC‐III Tg mice had slightly lowered lymph flow rates (approximately 20% reduced). When averaged over the 6‐h infusion period, the average lymph flow rate was slightly but significantly lower in apoCIII Tg mice than in WT mice (Fig. 2B).

**Figure 3:**
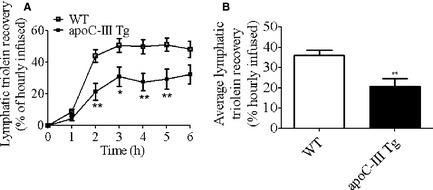
[^3^H]‐triolein recovery in lymph after duodenal infusion in WT and apoCIII Tg mice. Hourly (A) and average (B) lymphatic recovery of intraduodenally infused [^3^H]‐triolein in WT (*n* = 12) and apoC‐III Tg (*n* = 11) mice. Values are means ± SEM. *P < 0.05, **P < 0.01 versus WT mice.

To investigate whether apoC‐III overexpression leads to an enhanced rate of lipid transport, [^3^H]‐triolein secretion into lymph was measured during the 6‐h infusion period. As shown in Figure 3A, for both WT and apoC‐III Tg mice, [^3^H]‐triolein secretion into lymph reached a steady state level 3 h into the lipid infusion; however, the apoC‐III Tg mice had a striking reduction in lymphatic [^3^H]‐triolein at 2‐, 3‐, 4‐, and 5 h. When expressed as an average percentage of hourly lipid infused (Fig. 3B), WT mice secreted 43% more [^3^H]‐triolein into lymph than their apoC‐III Tg counterparts (*P* < 0.01). This decrease in lymphatic triolein transport was not coupled with steatorrhea (nor do apoC‐III Tg mice exhibit steatorrhea under chow conditions) (data not shown).

### Luminal content and distribution of radiolabeled lipid

**Figure 4:**
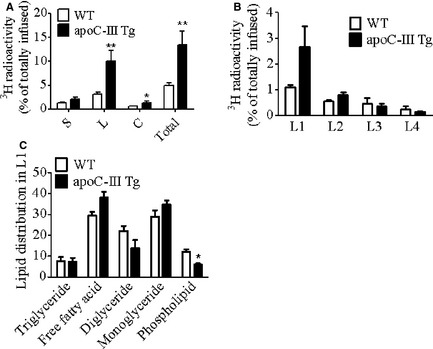
[^3^H]‐triolein recovery in gut lumen of WT and apoCIII Tg mice (A) Amount of [^3^H] radioactivity remaining in the lumen of stomach (S), entire small intestine (L), and colon (C) and their sum by the end of the 6‐h lipid infusion (*n* = 12 in WT,* n* = 11 in apoC‐III Tg). (B) Amount of [^3^H] radioactivity remaining in the lumen of equally divided four segments of the small intestine (L1–L4, representing the most proximal to most distal quarters, respectively) at the end of the 6‐h lipid infusion (*n* = 3). (C) Relative [^3^H] radioactivity distribution in different lipid species in the total lipid isolated from the first quarter of lumen (L1) (*n* = 3). Values are means ± SEM. *P < 0.05, **P < 0.01 versus WT mice.

After the hydrolysis of dietary TAG into FFA and MAG in the intestinal lumen, absorption by the intestine involves the uptake of that FFA and then its subsequent re‐esterification to TAG at the ER and transport into the lymph in chylomicrons. Therefore, in order to determine where the defect in lymphatic TAG secretion was occurring, we isolated the stomach, intestinal lumen, and colon of the apoC‐III Tg and WT mice after [^3^H]‐triolein infusion. As shown in Figure 4A, both apoC‐III Tg and WT mice had very small amounts (>2%) of the infused [^3^H]‐label in the stomach and colon, though the apoC‐III Tg mice had a small but significant increase in [^3^H]‐label in the colon. The largest difference was in the luminal compartment, where apoC‐III Tg mice retained 10.0% of the total [^3^H]‐dose, which was significantly higher than the amount of [^3^H]‐dose remaining in the lumen of the WT mice (Fig. 4A, *P* < 0.01).

As dietary TAG absorption occurs in a gradient along the length of the intestine, with greater absorption in the proximal quarter of the intestine, we investigated whether the defect in [^3^H]‐triolein uptake may occur in a specific location in the apoC‐III Tg mice. Indeed, as shown in Figure 4B, when we divided the intestine into four segments (1–4, proximal to distal), and then collected the luminal contents from those four segments (L1–L4), we found a twofold increase in the amount of [^3^H]‐label in the first quarter of the apoC‐III Tg intestine (the duodenum and proximal jejunum) when compared to the WT mice.

Since a known function of apoC‐III in the plasma is to inhibit lipoprotein lipase (LPL), we considered the possibility that overexpression of intestinal apoC‐III, may inhibit pancreatic lipase or colipase activity in the luminal compartment, thereby inhibiting the luminal hydrolysis of the infused [^3^H]‐triolein to FFA, which would prevent uptake by the enterocytes. However, when we analyzed the retained [^3^H]‐lipid from the L1 compartment of both the apoC‐III Tg and WT mice, we found that the percentage of FFA and MAG in the lumen of the apoC‐III Tg mice actually tended to be higher than in WT mice, though this difference did not reach significance (Figure 4C). This was accompanied by a difference in label incorporated into the phospholipid fraction. In addition, there was no difference in the amount of TAG remaining in the luminal compartment. Although not conclusive, these data suggest that the increased accumulation of [^3^H]‐label in the lumen of the apoC‐III Tg mice is not due to defect in lipolysis by pancreatic lipase.

### Mucosal content and distribution of radiolabeled lipids

**Figure 5:**
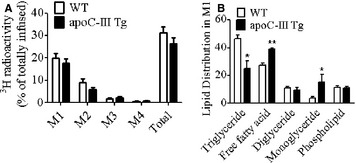
Lipid recovery in intestinal mucosa of WT and apoCIII Tg mice after duodenal infusion. (A) Amount of [^3^H]‐radioactivity remaining in the four segments of intestinal mucosa (M1–M4), proximal to distal, respectively, and their sum after a 6‐h lipid infusion (*n* = 12 in WT,* n* = 11 in apoC‐III Tg). (B) Relative [^3^H]‐radioactivity distribution in lipids in the first quarter of mucosa as determined by thin‐layer chromatography (*n* = 3). Values are means ± SEM. *P < 0.05, **P < 0.01 versus WT mice.

[^3^H]‐label retention in the intestinal enterocytes (mucosa) represents the lipid that was taken up from the lumen and is awaiting transport out of the intestine in chylomicrons. We hypothesized that there may be a defect in either the secretion of TAG in chylomicrons or in the re‐esterification of the absorbed FFA. Therefore, we isolated the mucosal lipid from the M1–M4 segments of the intestine (1–4 proximal to distal) at the end the 6‐h lipid infusion. As shown in Figure 5A, radioactive lipids were found mainly in the first quarter of the small intestinal mucosa (M1) in both WT and apoC‐III Tg animals, with a gradient decrease from proximal to distal. There were no significant differences between the apoC‐III Tg and WT mice in the amount of [^3^H]‐label retained in the mucosa. Surprisingly, however, when we determined the type of lipid retained in the M1 segment by thin‐layer chromatography, we found that the apoC‐III Tg mice had significantly less [^3^H]‐TAG and instead had an increase in mucosal FFA and MAG (Fig. 5B). Taken together with the increased FFA in the luminal compartment, this suggests that the apoC‐III Tg mice have a fundamental defect in lipid uptake and transport in the intestine.

## Discussion

Several genetically modified mouse models have been used to study the effect of apoC‐III on lipid metabolism in the circulation, and a detailed mechanism by which apoC‐III blocks hydrolysis and clearance of TRLs has been reported (Aalto‐Setälä et al. 1996; Mann et al. 1997; Altomonte et al. 2004; Cohn et al. 2004b; Gerritsen et al. 2005). In addition, emerging evidence in vitro suggests that apoC‐III plays an intracellular role in the liver to promote the assembly and secretion of VLDL (Cohn et al. 2004b; Sundaram et al. 2010a,b). Despite the crucial role of apoC‐III in determining plasma triglyceride levels and disease progression, very few studies have investigated the impact of apoC‐III in the intestine, where it is also expressed.

Using the lymph fistula mouse model, we were able to assess the apoC‐III effect on lipid absorption in a direct and quantitative manner since chylomicrons secreted by enterocytes are transported exclusively by the lymphatic system due to their large size precluding entry through the pores of the capillaries. Lipid absorption was calculated based on a known amount of infused radiolabeled lipid, thus avoiding the influence of circulating lipids and their metabolism on the quantification of secreted lipid in lymph. This is especially important for the study of apoC‐III, because apoC‐III has a major impact on circulating lipid metabolism. Using this model system, we discovered that overexpression of apoC‐III results in the inhibition of dietary TAG secretion from the intestine into lymph. ApoC‐III Tg mice also accumulate FFA in the intestinal lumen. These data suggest that apoC‐III plays a previously unknown role in lipid absorption, re‐esterification, and transport in the intestine. This role of apoC‐III in the intestine is unique from its role in both the plasma and the liver.

How might apoC‐III inhibit the uptake of FFA and MAG into enterocytes and its subsequent incorporation into TAG? On the basis of previous findings in the liver, which detail a role for intracellular apoC‐III in the liver TRL synthesis and secretion pathway (Sundaram et al. 2010a,b), we think it is likely that intracellular apoC‐III is also acting on the intestinal TRL synthesis and secretion pathway. The most likely site for this influence would be on the MGAT re‐esterification pathway, which predominates in the intestine. However, since the intestine expresses both MGAT and DGAT, and both of these enzymes are able to possess both the MGAT and DGAT activities, determining this mechanism is not necessarily straightforward.

Another possible explanation for the lack of TAG in the apoC‐III Tg mucosa could be due to a defect in trafficking of FFA to the ER for TAG synthesis. Since we observe an accumulation of FFA in the apoC‐III Tg mucosa coupled with a slight increase in the lumen, it is unlikely that there is a defect in FFA uptake into the cell (as would occur if there were changes to FFA transporters such as CD36). It would be more likely that the decrease in mucosal TAG in the apoC‐III Tg mice is due to a lack of conversion of mucosal FFA to fatty acyl‐CoA by acyl‐CoA synthetases (ACSL). Inhibition of this pathway would allow FFA to efflux back from the mucosa into the intestinal lumen before it could be used as a substrate for TAG synthesis.

Whether the existing hypertriglyceridemia in the apoC‐III Tg mice itself contributes to the decreased uptake and intestinal transport of TAG is an intriguing possibility. Since apoC‐III is expressed in both the intestine and the liver, it is unclear whether the inhibition of intestinal TAG secretion in the apoC‐III Tg mice is due directly to its expression in intestine, or indirectly due to expression in liver and subsequent stimulation of VLDL synthesis and plasma hypertriglyceridemia. TRL synthesis and secretion in the intestine and liver is a well‐coordinated and dynamic process between the postprandial and fasting states. The shift from postprandial to fasting involves factors including nutrient ingestion, glucose, insulin, the incretin hormones, in addition to neural signals. Potentially, apoC‐III secretion from the liver could be one of these factors.

Identifying whether intestinal or hepatic apoC‐III is responsible for the inhibition of intestinal TAG secretion is necessary to capitalize on this novel role for apoC‐III. Although we have not yet defined the mechanism by which overexpression of apoC‐III inhibits dietary fat absorption, we feel that this previously unknown intestinal role for apoC‐III may be of significant importance to the dietary fat absorption pathway. This is an important additional role for apoC‐III, a known modulator of plasma lipid levels and cardiovascular disease. In summary, this study reveals a novel role for apoC‐III in decreasing intestinal TAG transport into lymph distinct from its extracellular roles in plasma on lipoprotein lipase, and its intracellular role in hepatic VLDL synthesis and secretion. Given the important role of apoC‐III in modulating plasma TAG levels, this novel role in the intestine will be critical in order to exploit apoC‐III as a potential therapeutic target.

## Conflict of Interest

None declared.

## References

[phy2247-bib-0001] Aalto‐Setala, K. , E. A. Fisher , X. Chen , T. Chajek‐Shaul , T. Hayek , R. Zechner , et al. 1992 Mechanism of hypertriglyceridemia in human apolipoprotein (apo) CIII transgenic mice Diminished very low density lipoprotein fractional catabolic rate associated with increased apo CIII and reduced apo E on the particles. J. Clin. Invest. 90:1889–1900. 1430212 10.1172/JCI116066 PMC443250

[phy2247-bib-0002] Aalto‐Setälä, K. , P. H. Weinstock , C. L. Bisgaier , L. Wu , J. D. Smith , and J. L. Breslow . 1996 Further characterization of the metabolic properties of triglyceride‐rich lipoproteins from human and mouse apoC‐III transgenic mice. J. Lipid Res. 37:1802–1811. 8864964

[phy2247-bib-0003] Altomonte, J. , L. Cong , S. Harbaran , A. Richter , J. Xu , M. Meseck , et al. 2004 Foxo1 mediates insulin action on apoC‐III and triglyceride metabolism. J. Clin. Invest. 114:1493–1503. 15546000 10.1172/JCI19992 PMC525736

[phy2247-bib-0004] Blankenhorn, D. H. , P. Alaupovic , E. Wickham , H. P. Chin , and S. P. Azen . 1990 Prediction of angiographic change in native human coronary arteries and aortocoronary bypass grafts Lipid and nonlipid factors. Circulation 81:470–476. 2404631 10.1161/01.cir.81.2.470

[phy2247-bib-0005] Booth, C. C. , D. Alldis , and A. E. Read . 1961 Studies on the site of fat absorption. Gut 2:168–174. 18668743 10.1136/gut.2.2.168 PMC1413253

[phy2247-bib-0006] Brown, W. V. , R. I. Levy , and D. S. Fredrickson . 1969 Studies of the proteins in human plasma very low density lipoproteins. J. Biol. Chem. 244:5687–5694. 4981584

[phy2247-bib-0007] Cohn, J. S. J. , M. Tremblay , R. Batal , H. Jacques , C. Rodriguez , G. Steiner , et al. 2004a Increased apoC‐III production is a characteristic feature of patients with hypertriglyceridemia. Atherosclerosis 177:137–145. 15488876 10.1016/j.atherosclerosis.2004.06.011

[phy2247-bib-0008] Cohn, J. S. , B. W. Patterson , K. D. Uffelman , J. Davignon , and G. Steiner . 2004b Rate of production of plasma and very‐low‐density lipoprotein (VLDL) apolipoprotein C‐III is strongly related to the concentration and level of production of VLDL triglyceride in male subjects with different body weights and levels of insulin sensitivity. J. Clin. Endocrinol. Metab. 89:3949–3955. 15292332 10.1210/jc.2003-032056

[phy2247-bib-0009] Coleman, R. 2004 Enzymes of triacylglycerol synthesis and their regulation. Prog. Lipid Res. 43:134–176. 14654091 10.1016/s0163-7827(03)00051-1

[phy2247-bib-0010] Folch, J. , M. Lees , and G. H. Sloane Stanley . 1957 A simple method for the isolation and purification of total lipides from animal tissues. J. Biol. Chem. 226:497–509. 13428781

[phy2247-bib-0011] Fredenrich, A. , L. M., Giroux , M., Tremblay , L., Krimbou , J., Davignon , and J. S. Cohn . 1997 Plasma lipoprotein distribution of apoC‐III in normolipidemic and hypertriglyceridemic subjects: comparison of the apoC‐III to apoE ratio in different lipoprotein fractions. J. Lipid Res. 38:1421–1432. 9254067

[phy2247-bib-0012] Gangabadage, C. S. , J. Zdunek , M. Tessari , S. Nilsson , G. Olivecrona , and S. S. Wijmenga . 2008 Structure and dynamics of human apolipoprotein CIII. J. Biol. Chem. 283:17416–17427. 18408013 10.1074/jbc.M800756200

[phy2247-bib-0013] Gerritsen, G. , P. C. N. Rensen , K. E. Kypreos , V. I. Zannis , L. M. Havekes , and K. Willems van Dijk . 2005 ApoC‐III deficiency prevents hyperlipidemia induced by apoE overexpression. J. Lipid Res. 46:1466–1473. 15863838 10.1194/jlr.M400479-JLR200

[phy2247-bib-0014] Ginsberg, H. N. and W. V. Brown . 2011 Apolipoprotein CIII: 42 years old and even more interesting. Arterioscler. Thromb. Vasc. Biol. 31:471–473. 21325666 10.1161/ATVBAHA.110.221846

[phy2247-bib-0015] Guo, Q. , R. K. Avramoglu , and K. Adeli . 2005 Intestinal assembly and secretion of highly dense/lipid‐poor apolipoprotein B48‐containing lipoprotein particles in the fasting state: evidence for induction by insulin resistance and exogenous fatty acids. Metabolism 54:689–697. 15877301 10.1016/j.metabol.2004.12.014

[phy2247-bib-0016] Haddad, I. A. , J. M. Ordovas , T. Fitzpatrick , and S. K. Karathanasis . 1986 Linkage, evolution, and expression of the rat apolipoprotein A‐I, C‐III, and A‐IV genes. J. Biol. Chem. 261:13268–13277. 3020028

[phy2247-bib-0017] Hokanson, J. E. , G. L. Kinney , S. Cheng , H. A. Erlich , A. Kretowski , and M. Rewers . 2006 Susceptibility to type 1 diabetes is associated with ApoCIII gene haplotypes. Diabetes 55:834–838. 16505251 10.2337/diabetes.55.03.06.db05-1380

[phy2247-bib-0018] Ito, Y. , N. Azrolan , A. O'Connell , A. Walsh , and J. Breslow . 1990 Hypertriglyceridemia as a result of human apo CIII gene expression in transgenic mice. Science 249:790–793. 2167514 10.1126/science.2167514

[phy2247-bib-0019] Kinnunen, P. K. and C. Ehnolm . 1976 Effect of serum and C‐apoproteins from very low density lipoproteins on human postheparin plasma hepatic lipase. FEBS Lett. 65:354–357. 182536 10.1016/0014-5793(76)80145-7

[phy2247-bib-0020] Kohan, A. B. , F. Wang , X. Li , S. Bradshaw , Q. Yang , J. L. Caldwell , et al. 2012 Apolipoprotein A‐IV regulates chylomicron metabolism‐mechanism and function. Am. J. Physiol. Gastrointest. Liver Physiol. 302:G628–G636. 22207575 10.1152/ajpgi.00225.2011 PMC3311309

[phy2247-bib-0021] Kumar, N. S. and C. M. Mansbach II . 1999 Prechylomicron transport vesicle: isolation and partial characterization. Am. J. Physiol. 276:G378–G386. 9950811 10.1152/ajpgi.1999.276.2.G378

[phy2247-bib-0022] Landis, B. A. , F. S. Rotolo , W. C. Meyers , A. B. Clark , and S. H. Quarfordt . 1987 Influence of apolipoprotein E on soluble and heparin‐immobilized hepatic lipase. Am. J. Physiol. 252:G805–G810. 3591946 10.1152/ajpgi.1987.252.6.G805

[phy2247-bib-0023] Lee, S.‐J. , H. Campos , L. A. Moye , and F. M. Sacks . 2003 LDL containing apolipoprotein CIII is an independent risk factor for coronary events in diabetic patients. Arterioscler. Thromb. Vasc. Biol. 23: 853–858. 12637336 10.1161/01.ATV.0000066131.01313.EB

[phy2247-bib-0024] Lenich, C. , P. Brecher , S. Makrides , A. Chobanian , and V. I. Zannis . 1988 Apolipoprotein gene expression in the rabbit: abundance, size, and distribution of apolipoprotein mRNA species in different tissues. J. Lipid Res. 29:755–764. 3171395

[phy2247-bib-0025] Lo, C. M. , B. K. Nordskog , A. M. Nauli , S. Zheng , S. B. Vonlehmden , Q. Yang , et al. 2008 Why does the gut choose apolipoprotein B48 but not B100 for chylomicron formation? Am. J. Physiol. Gastrointest. Liver Physiol. 294:G344–G352. 18006607 10.1152/ajpgi.00123.2007

[phy2247-bib-0026] Maeda, N. , H. Li , D. Lee , P. Oliver , S. H. Quarfordt , and J. Osada . 1994 Targeted disruption of the apolipoprotein C‐III gene in mice results in hypotriglyceridemia and protection from postprandial hypertriglyceridemia. J. Biol. Chem. 269:23610–23616. 8089130

[phy2247-bib-0027] Mann, C. J. , A. A. Troussard , F. T. Yen , N. Hannouche , J. Najib , J. C. Fruchart , et al. 1997 Inhibitory effects of specific apolipoprotein C‐III isoforms on the binding of triglyceride‐rich lipoproteins to the lipolysis‐stimulated receptor. J. Biol. Chem. 272:31348–31354. 9395464 10.1074/jbc.272.50.31348

[phy2247-bib-0028] Mansbach, C. M., II , and F. Gorelick . 2007 Development and physiological regulation of intestinal lipid absorption II Dietary lipid absorption, complex lipid synthesis, and the intracellular packaging and secretion of chylomicrons. Am. J. Physiol. Gastrointest. Liver Physiol. 293:G645–G650. 17627968 10.1152/ajpgi.00299.2007

[phy2247-bib-0029] McConathy, W. J. , J. C. Gesquiere , H. Bass , A. Tartar , J. C. Fruchart , and C. S. Wang . 1992 Inhibition of lipoprotein lipase activity by synthetic peptides of apolipoprotein C‐III. J. Lipid Res. 33:995–1003. 1431591

[phy2247-bib-0030] Qu, S. , G. Perdomo , D. Su , F. M. D'Souza , N. S. Shachter , and H. H. Dong . 2007 Effects of apoA‐V on HDL and VLDL metabolism in APOC3 transgenic mice. J. Lipid Res. 48:1476–1487. 17438339 10.1194/jlr.M600498-JLR200 PMC2665252

[phy2247-bib-0031] Rahim, A. , E. Nafi‐valencia , S. Siddiqi , R. Basha , C. C. Runyon , and S. A. Siddiqi . 2012 Proteomic analysis of the very low density lipoprotein (VLDL) transport vesicles. J. Proteomics 75:2225–2235. 22449872 10.1016/j.jprot.2012.01.026 PMC3341533

[phy2247-bib-0032] Roggin, G. M. , F. L. Iber , and W. G. Linscheer . 1972 Intraluminal fat digestion in the chronic alcoholic. Gut 13:107–111. 5045703 10.1136/gut.13.2.107 PMC1412067

[phy2247-bib-0033] Sacks, F. M. , P. Alaupovic , L. A. Moye , T. G. Cole , B. Sussex , M. J. Stampfer , et al. 2000 VLDL, apolipoproteins B, CIII, and E, and risk of recurrent coronary events in the Cholesterol and Recurrent Events (CARE) trial. Circulation 102:1886–1892. 11034934 10.1161/01.cir.102.16.1886

[phy2247-bib-0034] Schonfeld, G. , P. K. George , J. Miller , P. Reilly , and J. Witztum . 1979 Apolipoprotein C‐II and C‐III levels in hyperlipoproteinemia. Metabolism 28:1001–1010. 226830 10.1016/0026-0495(79)90004-0

[phy2247-bib-0035] Sehayek, E. and S. Eisenberg . 1991 Mechanisms of inhibition by apolipoprotein C of apolipoprotein E‐dependent cellular metabolism of human triglyceride‐rich lipoproteins through the low density lipoprotein receptor pathway. J. Biol. Chem. 266:18259–18267. 1917954

[phy2247-bib-0036] Sundaram, M. , S. Zhong , M. Bou Khalil , P. H. Links , Y. Zhao , J. Iqbal , et al. 2010a Expression of apolipoprotein C‐III in McA‐RH7777 cells enhances VLDL assembly and secretion under lipid‐rich conditions. J. Lipid Res. 51:150–161. 19622837 10.1194/jlr.M900346-JLR200 PMC2789775

[phy2247-bib-0037] Sundaram, M. , S. Zhong , M. Bou Khalil , H. Zhou , Z. G. Jiang , Y. Zhao , et al. 2010b Functional analysis of the missense APOC3 mutation Ala23Thr associated with human hypotriglyceridemia. J. Lipid Res. 51:1524–1534. 20097930 10.1194/jlr.M005108 PMC3035516

[phy2247-bib-0038] Wu, A. L. and H. G. Windmueller . 1978 Identification of circulating apolipoproteins synthesized by rat small intestine in vivo. J. Biol. Chem. 253:2525–2528. 632283

[phy2247-bib-0039] Xiao, C. , J. Hsieh , K. Adeli , and G. F. Lewis . 2011 Gut‐liver interaction in triglyceride‐rich lipoprotein metabolism. Am. J. Physiol. Endocrinol. Metab. 301:E429–E446. 21693689 10.1152/ajpendo.00178.2011

[phy2247-bib-0040] Zheng, C. , C. Khoo , K. Ikewaki , and F. M. Sacks . 2007 Rapid turnover of apolipoprotein C‐III‐containing triglyceride‐rich lipoproteins contributing to the formation of LDL subfractions. J. Lipid Res. 48:1190–1203. 17314277 10.1194/jlr.P600011-JLR200

[phy2247-bib-0041] Zheng, C. , C. Khoo , J. Furtado , and F. M. Sacks . 2010 Apolipoprotein C‐III and the metabolic basis for hypertriglyceridemia and the dense low‐density lipoprotein phenotype. Circulation 121:1722–1734. 20368524 10.1161/CIRCULATIONAHA.109.875807 PMC3153990

